# Family physicians’ questions about the COVID-19 pandemic: a content analysis of 2,272 helpline calls

**DOI:** 10.1186/s12875-023-02147-w

**Published:** 2023-09-20

**Authors:** Allan McDougall, Jacqueline H. Fortier, Cathy Zhang, Caroline Ehrat, Kerri Best, Heather Blois, Gary Garber

**Affiliations:** 1 Faculty of Education, University of Ottawa, Ottawa, ON Canada; 2 The Canadian Medical Protective Association, Ottawa, ON Canada; 3 Department of Medicine, University of Ottawa, Ottawa Hospital Research Institute, 501 Smyth Rd, Ottawa, ON K1H 8L6 Canada; 4 Department of Medicine, University of Toronto, Toronto, ON Canada

**Keywords:** COVID-19, Family medicine, Physician experiences, Content analysis

## Abstract

**Background:**

During the COVID-19 pandemic, family physicians faced challenges including travel restrictions for patients, lockdowns, diagnostic testing delays, and changing public health guidelines. Given that 95% of Canadian physicians are members of the Canadian Medical Protective Association (CMPA), the CMPA’s telephone helpline — which offers peer-to-peer support — provides valuable insights into family physicians’ experiences during the pandemic.

**Methods:**

We used a content analysis approach to identify and understand family physicians’ questions and concerns related to the COVID-19 pandemic expressed during calls to the Canadian Medical Protective Association (CMPA) telephone helpline. Calls were classified with preliminary codes and subsequently organized into themes. We collected aggregated data on calls, including province, call date, and whether the physician self-identified having hospital-based activities as part of their practice. Findings from the analysis were explored alongside family physician calls per month (call volume).

**Results:**

Between 01 and 2020 and 31 December 2021, 2,272 family physician calls related to the pandemic were included for content analysis. We identified six major themes across these calls: challenging patient interactions; COVID-related care; the impact of the pandemic on the healthcare system; virtual care; physician obligations and rights; and public health matters. COVID-related call volumes were highest early in the pandemic especially among physicians without major hospital affiliation when family physicians practiced with little guidance on how to balance patient care and scarce resources in the face of a novel pandemic.

**Conclusions:**

This research provides unique insight on the effects the COVID-19 pandemic had on family medicine in Canada. These results provide insights on the needs and information gaps of family physicians in a public health crisis and can inform preparedness efforts by public health agencies, professional organizations, educators, and practitioners.

**Supplementary Information:**

The online version contains supplementary material available at 10.1186/s12875-023-02147-w.

## Background

Throughout the coronavirus disease 2019 (COVID-19) pandemic, family physicians faced challenges associated with travel restrictions for patients, lockdowns, quarantine, gaps in testing infrastructure, lack of PPE supply, and calls to provide clinical services to testing centres, vaccination sites, and long-term care settings [1–3]. In nearly every aspect of their work, family physicians felt pressure as a result of having to integrate evolving public health guidelines around social distancing and infection control measures such as masks and hand hygiene [4]. Furthermore, family physicians reported experiencing emotional and mental health challenges while managing the unique demands of providing care during the pandemic [5, 6]. COVID-19 has also led to substantial changes to the practice of medicine, including the need to conduct assessments and provide health education virtually, and the need to provide testing and immunizations with distancing measures in place [7].

In Canada, approximately 95% of practicing physicians are members of the Canadian Medical Protective Association (CMPA), a national mutual defense organization for physicians. The 105,000 members of the CMPA have access to a telephone helpline where they can call with concerns or questions that have emerged in their practice. The helpline primarily addresses concerns and questions related to medical liability and risk management. Starting in early 2020, CMPA physician advisors began providing peer-to-peer support conversations related to the COVID-19 pandemic, reflecting physicians’ concerns about their patients and their clinical practice [8]. We hypothesized that information related to calls to this helpline would offer important insights into family physicians’ experiences during the unprecedented COVID-19 pandemic.

In Canada, family medicine is a primary care specialty that encompasses a broad range of medical knowledge and skills. Family physicians provide medical care for patients of all ages and across the care spectrum. They are also the primary path through which patients access specialist care, based on a family physician’s referral. Given this broad scope of practice, family physicians are uniquely positioned to provide insights into the challenges and experiences of primary healthcare providers during the COVID-19 pandemic. The purpose of this study was to evaluate the COVID-related questions and concerns from family physicians and to explore how patterns of calls changed as the pandemic evolved.

## Methods

### Data source and extraction criteria

When physicians contact the CMPA, they speak with a physician advisor who addresses their questions. Calls to the CMPA helpline are not recorded, but information about the purpose of the call and the information exchanged is captured in short memos following consistent annotation procedures, which have been previously described [8, 9]. These memos are part of the CMPA’s national repository of routinely collected data. Although the memos’ contents vary, they are intended to document the physician’s questions or concerns and summarize the advice given by the physician advisor.

Throughout the COVID-19 pandemic, physician advisors applied a tag to calls related to the pandemic. Our analysis included de-identified COVID-related memos for calls from family physicians placed between 01 and 2020 and 31 Dec 2021. We delineated the pandemic into Canada’s first, second, third, and fourth waves: 01 March 2020–31 August 2020; 01 September 2020–28 February 2021; 01 March 2021–30 June 2021; and 01 July 2021–31 December 2021.

We calculated numbers of calls and rates of calls per 1000 family physicians. For each memo, our database included date of the call, province of practice, and postal code. We also collected whether callers self-identified as having a hospital-based activities as a substantial part of their practice (i.e., their practice includes anesthesia, emergency medicine, surgery, or obstetrics). Calls from the Atlantic provinces (New Brunswick, Newfoundland and Labrador, Prince Edward Island, and Nova Scotia) and the territories (Nunavut, Northwest Territories, and Yukon Territory) were grouped in our reporting due to small call numbers. Call memos were imported into a database for coding. Data analyses was conducted using SAS software version 9.4 [10]. A list of extracted variables can be found in Supplemental Table 1.

### Data analysis

Our analysis of family physicians’ concerns used content analysis and followed published guidance for thematic analysis of text-based data [11, 12]. Team members reviewed each memo, seeking to answer the question *what are the physician’s concerns expressed during the call?* Using Microsoft Excel, coding proceeded in three phases. First, three team members (JF, AM, CZ) synchronously reviewed a random sample of 50 calls to generate a list of physician concerns. As an example, a call from a physician concerned about a lack of masks for healthcare workers was coded *PPE*. We allowed that a memo may have more than one code, for example a call concerning a patient stranded in another country due to travel restrictions was coded *Virtual health* and *Cross-border care*.

**Fig. 1:**
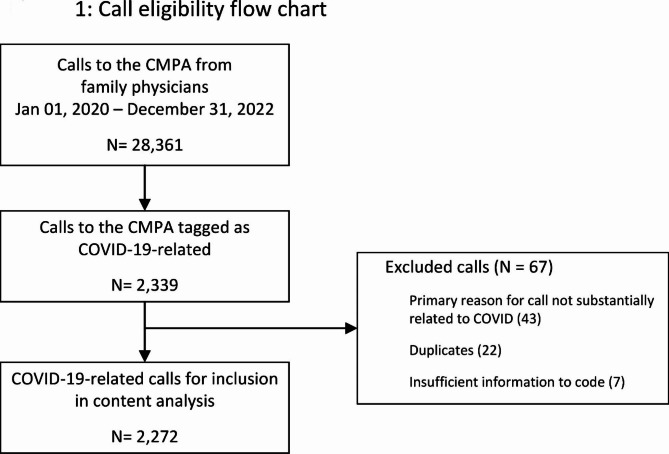
Call eligibility flow chart

Next, two team members (JF, AM) served as primary coders and asynchronously reviewed and coded an additional sample of 60 calls using the identified codes. We used Fleiss’ kappa to calculate inter-rater reliability, with a target agreement between 0.61 and 0.80, considered “substantial agreement.” [13] After this calibration exercise, the coders individually coded the remaining calls. Frequent coding meetings allowed team members to discuss consistent coding and any emergent codes. We included all call memos dated between 01 January 2020 and 31 December 2021 and tagged as related to the pandemic. The primary coders discussed any calls that might require exclusion from the dataset (see Fig. 1). Call memos were excluded if they were duplicate calls that were follow-ups on an earlier matter, contained insufficient information for coding, or if the primary reason for the call was not substantially related to the pandemic. In the event of a discrepancy, a third team member (GG) was available to facilitate consensus. Once coding was complete, the entire study team worked to group the codes into higher-order themes over the course of 3 team meetings [14].

To explore whether affiliation with a hospital impacted the volume or type of family physicians’ concerns, we used Wilcoxon signed rank sum test to compare COVID-related call volume by theme between family physicians who self-identified as having a hospital-affiliated practice and those who did not.

## Results

**Fig. 2:**
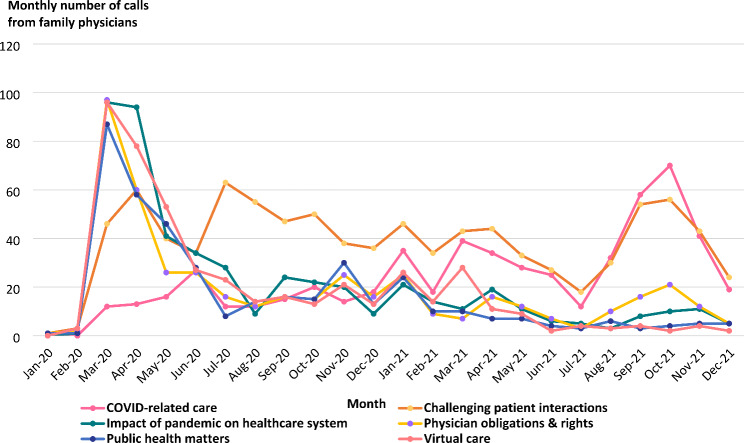
COVID-19-related family physician call volume trends by theme (n = 2,272)

From 01 to 2020 to 31 December 2021, the CMPA received 28,361 calls from family physicians, of which 2,272 were related to COVID-19 (Fig. 1). A rapid increase in weekly COVID-related call volume occurred in March 2020, peaking at 113 calls (39.5% of all calls from family physicians) the week of March 22, 2020 (Fig. 2). While we observed a sudden growth in COVID-19-related calls from family physicians in the early days of the pandemic, we did not see an overall increase in call volume from this group when compared to pre-pandemic levels; the increase in pandemic-related calls was offset by a decrease in non-COVID-related calls. As COVID-19-related calls decreased over time, calls about other medico-legal matters increased.

**Fig. 3:**
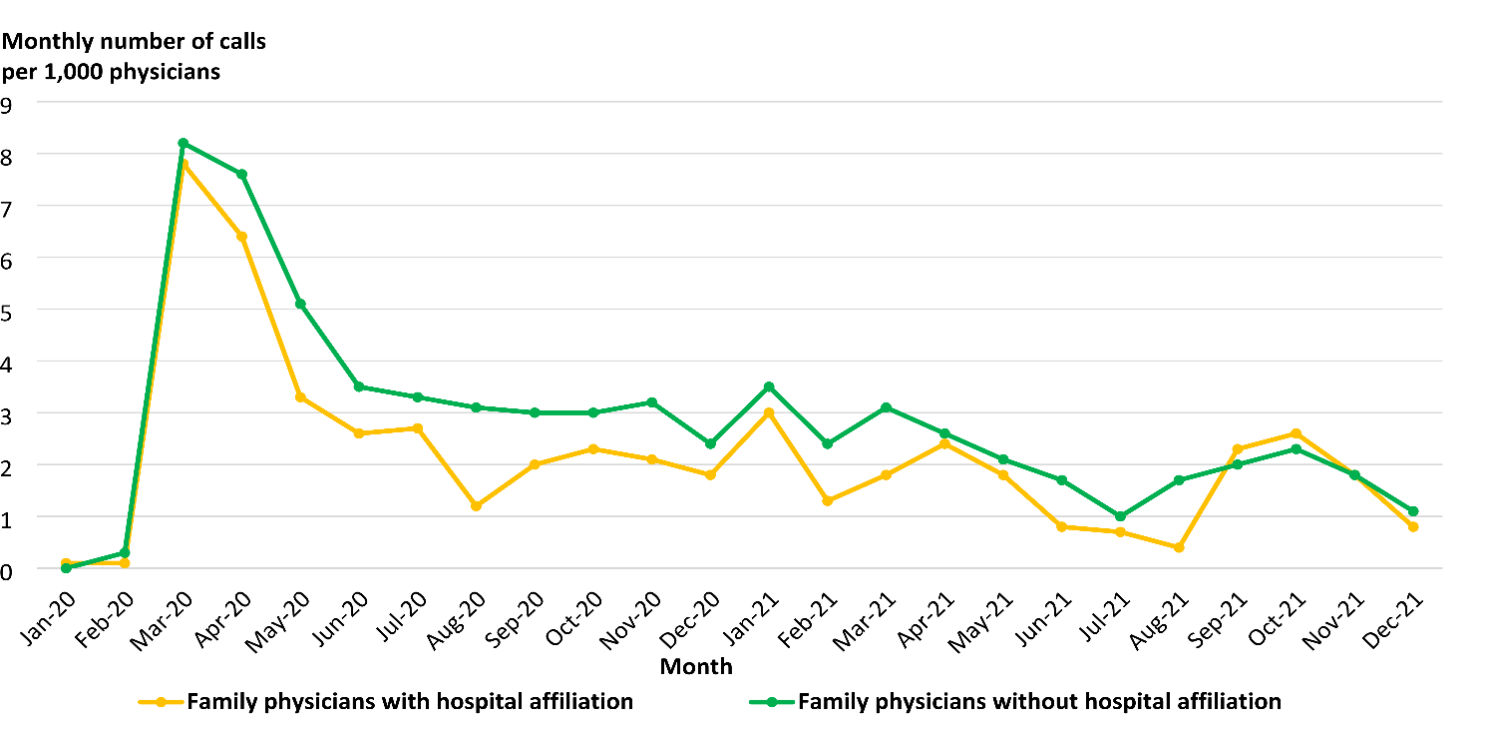
COVID-19-related call volume family physicians with hospital affiliation (n = 2,272)

We observed that family physicians who self-identified having hospital-based activities as part of their practice had lower call volume rates throughout the pandemic than family physicians who were not affiliated with a hospital. (Fig. 3)

### Themes

**Fig. 4:**
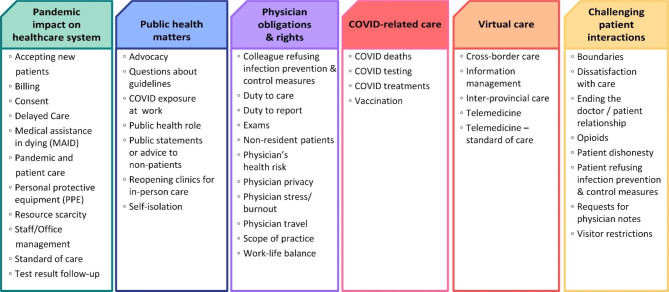
Themes and codes used to classify concerns raised by family physicians during COVID-19 related calls to the CMPA

A total of 45 codes were identified through the process of content analysis, which were grouped into 6 higher-level themes: Challenging patient interactions, COVID-related care, Pandemic’s impact on the healthcare system, Virtual care, Physician obligations & rights, and Public health matters (Fig. 4). A list of the 45 codes can be found in Supplemental Table 2. On average, coders identified 1.6 codes (SD 0.6) per call. References to physicians’ calls were re-written as mock scenarios for the purpose of reporting our results. These scenarios capture the original concerns but replace identifiable case characteristics such as names and places to protect the privacy of family physicians and their patients. Developing scenarios like these is an established practice in some approaches to qualitative data analysis [27].

### Challenging patient interactions

The most coded theme was challenging patient interactions (N = 925). Of these calls, 447 were seeking advice related to patients’ requests for COVID-related doctors’ notes for a variety of reasons. These requests involved mask exemptions, returning to / staying home from work or school, documentation related to quarantine and vaccine exemption. As some memos described, patients with comorbidities were required by public health and employer guidelines to provide a physician letter exempting their patient from returning to work:

*Dr. A’s patient is a school bus driver over 65, whose employer wishes them to return to work. Dr. A assesses that the patient’s other health conditions put them at risk for COVID-19. The patient has requested a physician letter stating that it is not safe for the patient to work. Dr. A wishes to discuss wording for this letter.*

Family physicians also sought guidance for declining patient requests for a physician’s letter. Some memos described call regarding patients requesting mask deferrals:

*Dr. C called regarding a patient who has indicated they have a breathing condition exacerbated by masks. Dr. C is unaware of any history of a breathing condition but is concerned the patient will become upset at having their request declined.*

Other challenging interactions involved 219 calls relating to patients who refused to comply with infection prevention and control measures. A memo involving a call with Dr. E exemplifies such a compliance call:

*Dr. E called with questions regarding how to respond to a long-time patient who presented to the clinic and refused to wear a mask. Dr. E is unclear how to proceed providing care for an unmasked patient given the potential risk to their staff and other patients.*

### COVID-related care

The next most common call topic for family physicians was COVID-related care (N = 570 calls). 306 of these calls related to vaccines and immunization. The volume of monthly calls about COVID-related care rose from 8 calls per month (SD 7.7) during the second wave, then 20 calls per month (SD 2.9) during the third wave, and 32 calls per month (SD 21.0) during the fourth wave, as vaccines became more widely available (Fig. 2). Some physicians shared situations about colleagues who refused vaccination:

*Dr. G’s patient works in a nursing home and has requested a medical exemption for COVID-19 vaccination. The patient reported having a fever after a previous vaccine. Dr. G refused because of a lack of indication. The patient has threatened a regulatory college complaint and ended the appointment. Dr. G called to discuss the situation.*

COVID-related care calls also included issues around COVID-19 testing. As described in a scenario regarding a testing-related call from a physician:

*Dr. I called about a patient requesting a COVID-19 test. The patient does not meet local eligibility criteria but has requested one so that they can feel comfortable visiting an immunocompromised family member. Dr. I is unsure how to proceed.*

#### Pandemic’s impact on the healthcare system

A total of 502 calls contained questions related to the impact of the pandemic on the healthcare system. Many family physicians expressed concerns over changes to how they manage their offices or staff during the pandemic. Some memos recounted physician calls about staffing concerns:

*Dr K has called with questions related to staff who have expressed concern coming to work during the pandemic. Dr K is concerned the clinic does not have adequate resources and technology for staff to work effectively off site.*

Further, family physicians called with questions related to providing safe care in the face of new restrictions and resource shortages., such as this scenario about a physician member of a large family medicine clinic:

*Dr M is a member of a family health team and the physicians are working through a lack of guidance on ensuring consistent well-baby care during the pandemic, particularly given limitations on physical exams. The clinic has also run out of medical masks and they are unclear when more will become available.*

#### Virtual care

Virtual care calls (N = 466) were focused on the pivot to providing clinical care via telemedicine and other electronic platforms. These calls included concerns about maintaining the standard of care given restrictions on in-person assessments:

*Dr P called with questions about ensuring she is providing appropriate care as her clinic shifts to virtual care. She is concerned about how to care for patients in her practice who may be difficult or impossible to adequately assess via telemedicine, including patients who struggle to communicate in English.*

Other calls related to virtual care involved ensuring respect for patients’ privacy and collection of health information. As described in this scenario about a privacy matter:

*Dr R’s clinic does not have a secure platform to facilitate virtual care visits and she has been communicating with patients via email. She called to discuss ensuring privacy until her clinic implements telemedicine software at the end of the month.*

Family physicians also called about providing care for patients across provincial and international borders. Some memos related situations involving stranded patients:

*Dr T’s patient has unfortunately been stranded abroad after COVID restrictions were put in place preventing international travel. The patient has requested medication refills, but Dr. T is not licensed to practice in that country. Dr. T wishes to ensure she responds appropriately.*

### Physician obligations & rights

Family physicians also called regarding their professional obligations and rights (N = 455 calls). Many family physicians called with questions regarding their duty of care, especially in the context of balancing patient care with risks to their own health and the health of staff, other patients and their households. Some memos recounted physicians wishing to discuss their obligation to take shifts at local intensive care units (ICUs):

*Dr S. has been asked to fill shifts at the local hospital’s intensive care unit. He wished to discuss whether he can be forced to work in the ICU given concerns about spreading the virus to his family.*

This theme also included family physicians calling to express concern about providing care while struggling with issues of staffing shortages, stress, burnout, and challenging work-life balance:

*Dr. V works as a hospitalist in a rural community hospital. The hospital has been faced with shortages of physicians to provide care and Dr. V is struggling with burnout. He wished to discuss the implications of taking a health leave.*

### Public health matters

Calls where family physicians described balancing system-level issues in the context of the pandemic were labeled as public health matters (N = 405). Many family physicians called with queries regarding guidelines from public health agencies:

*Dr. D’s municipality has not issued a mask mandate. Dr. D called to discuss issues related to implementing a mask-wearing requirement in their clinic.*

Another portion of these calls concerned requests for family physicians to speak publicly about the COVID-19 pandemic:

*Dr. B has been asked to speak to a reporter from a local newspaper about the impact of the COVID-19 pandemic on seniors in her community. She has never been interviewed before and would like some advice about how to proceed.*

## Discussion

During a 24-month period, family physicians called the CMPA over 2,200 times with concerns related to the COVID-19 pandemic. We identified six major themes across family physicians’ calls: the impact of the pandemic on the healthcare system; challenging patient interactions; public health matters; physician obligations and rights; virtual care; and COVID-related care. Calls to the helpline reflected key areas where family physicians faced unique pressures on the front-line during the pandemic.

Despite an abundance of recent research reporting on the striking shift to virtual care and its impact on primary care patterns, [15–19] we find it noteworthy that family physicians more frequently called with concerns related to challenging patient interactions throughout the pandemic. Requests for a physician’s note or medical documentation was the most frequently coded concern for family physicians (N = 447; 19.7% of calls). Research exploring physician communication around providing physician’s notes in primary care prior to the pandemic has cited a lack of guidance for both physicians and patients [20–22].

We noted a striking increase in calls related to vaccination when COVID-19 vaccines became more widely available for the public. These data, along with the increase in calls at the beginning of the pandemic, suggest that public health crises are accompanied by increases in questions and concerns amongst family doctors and suggests a communication gap between public health pronouncements and ability to implement at the primary care level. Strengthening communication between public health authorities and primary care providers could help reduce this gap and improve the implementation of public health measures in future crises.

Another potential contributor to the reduction in call volumes after the initial peak was increasingly available information and guidance as the pandemic progressed. In response to increasing call, the CMPA developed an online COVID-19 hub with answers to frequently asked questions to support members; this site has been accessed over 200,000 times [23]. Other medical associations and medical regulatory authorities developed similar online resources. The availability of this information from reliable sources likely reduced the number of calls to the physician helpline over time.

Family physicians whose practices included hospital-based activities such as surgery, obstetrics or anesthesia had lower call rates across each theme group. We propose further research exploring whether this difference indicates that hospital affiliation provides the added benefit of guidance, such as occupational health and safety offices, institutional interpretation of public health measures, and access to hospital legal counsel. Relatedly, we note the possibility that family physicians without a hospital-based practice may have had less support, resulting in the need to reach out to physician services organization with their concerns. This finding underscores the importance of access to supportive knowledge organizations such as medical associations and physician organizations. To better support physicians without hospital affiliations, future efforts could involve expanding support networks and resources tailored to their specific needs and circumstances.

Our work expands on existing research exploring physician concerns during the COVID-19 pandemic. A recent US-based analysis explored summaries of physicians’ calls to their medical liability services provider during the early months of COVID-19 [24, 25]. Many of these calls related to operational issues, including information on liability waivers. In contrast, our findings provided an expanded landscape of COVID-19 concerns with a focus on family physicians. Our results provide richer detail on key issues in family medicine, such as potential complications in patient communication that contributed to some family doctors reaching out to a helpline. These insights can help inform the development of tailored resources and support systems to address the specific challenges faced by family physicians during public health crises.

### Limitations

This research is not without limitations. We acknowledge that these data present a snapshot of an evolving pandemic. We further acknowledge these data lack in-depth information about important factors of each family physician’s practice, such as socioeconomic demographics. Our findings are based on a sample of CMPA members and the call data may not be representative of all CMPA members or all physicians practicing in Canada. Our assessment of hospital affiliation is based on physician self-identified their type of work to the CMPA. The self-reported nature of hospital affiliation might lead to underreporting or overreporting, which could affect our interpretation of the findings.

The use of aggregated data may limit our ability to identify specific associations between individual factors and call themes. Additionally, aggregated data may not capture important nuances or variations within the data. These limitations could potentially limit the generalizability of the results and our understanding of the true nature of the concerns raised by family physicians. Further, we acknowledge that multiple errors and biases may interfere with routine data collection and processing (e.g., undetected data linkage issues) [26].

## Conclusions

Family doctors called the CMPA’s helpline with a variety of COVID-related concerns. By identifying family physicians’ evolving concerns, we hope our results can inform targeted strategies for educational initiatives and future research to continue to support the crucial work of primary care. Family physicians’ pandemic-related questions were diverse and thoughtful, serving as a lasting testament to the versatility and resilience of front-line physicians persevering through the COVID pandemic. Our findings underscore the importance of providing timely, accessible information and support resources to family physicians during public health crises. By learning from these family physicians’ experiences, we can better prepare healthcare systems, physicians, and public health organizations to respond effectively to future challenges and ensure the continued delivery of high-quality primary care.

### Electronic supplementary material

Below is the link to the electronic supplementary material.

Supplementary Material 1

Supplementary Material 2

## Data Availability

The data that support the findings of this study are available from the Canadian Medical Protective Association (CMPA) but restrictions apply to the availability of these data, which were used under license for the current study, and so are not publicly available. Data are however available from the corresponding author upon reasonable request and with permission of the CMPA.

## Abbreviations

CMPA: Canadian Medical Protective Association
COVID-19: Coronavirus disease of 2019
