# Supplementary material for: Family physicians’ questions about the COVID-19 pandemic: a content analysis of 2,272 helpline calls
Source: BMC Prim Care. 2023 Sep 20;24:192. doi: 10.1186/s12875-023-02147-w (PMC10510291; doi:10.1186/s12875-023-02147-w)
Supplement: Supplementary file 1 — Supplementary Material 1 [file 12875_2023_2147_MOESM1_ESM.docx]

## Supplemental Table 1: List of extracted variables

| **Variable** | **Definition** |
| --- | --- |
| Call data | |
| Date of call | The date of the member’s call |
| Call summary memo | The physician advisor’s memo summarizing the call |
| Caller membership data | |
| Province of practice | Physician caller’s province of practice |
| Postal code | Physician caller’s postal code |
| Content analysis | |
| Themes | Themes identified by content analysis (up to 4) |
