# Supplementary material for: Family physicians’ questions about the COVID-19 pandemic: a content analysis of 2,272 helpline calls
Source: BMC Prim Care. 2023 Sep 20;24:192. doi: 10.1186/s12875-023-02147-w (PMC10510291; doi:10.1186/s12875-023-02147-w)
Supplement: Supplementary file 2 — Supplementary Material 2 [file 12875_2023_2147_MOESM2_ESM.docx]

Supplemental Table 2: Content analysis theme and code definitions

|  |  |
| --- | --- |
| **Theme 1. COVID-related care:** Family physicians seeking guidance related to COVID-related patient care. | |
| **Code** | **Definition** |
| COVID deaths | The caller has concerns related to COVID deaths. |
| COVID testing | The caller has questions related to COVID testing |
| COVID treatments | The caller has concerns or questions about treatments for patients with COVID-19 (e.g. medications, proning, use of modified ventilators) |
| Vaccination | The caller has questions or concerns regarding COVID vaccination. |
| **Theme 2. Pandemic impact on healthcare system:** Family physicians seeking guidance regarding the pandemic's impact on the health care system. | |
| **Code** | **Definition** |
| Accepting new patients | The caller has questions about accepting new patient referrals during the pandemic |
| Billing | The caller has questions or concerns about a billing matter related to the pandemic. |
| Consent | The caller has questions or concerns about changes to the process of obtaining and/or documenting consent from patients during the pandemic. |
| Delayed care | The caller is concerned about pandemic-related delays to a patient’s care |
| MAID | Calls related to the provision of medical assistance in dying (MAID) during the pandemic. |
| Pandemic and patient care | The caller has concerns about patient care decisions impacted by resource scarcity, care delays or other pandemic restrictions. |
| PPE | The caller has questions or concerns related to the use or availability of personal protective equipment (PPE). |
| Resource scarcity | The caller is calling about resource scarcities, including shortages of beds, equipment, staff, or procedural time. |
| Staff / office management | The caller has a concern related to their employee or some element of their office management during the pandemic (e.g., waiting room policies) |
| Test results follow up | The caller has concerns that test results won’t be followed up adequately due to COVID. |
| **Theme 3. Challenging patient interactions** Family physicians seeking guidance related to pandemic-related challenging patient interactions. | |
| **Code** | **Definition** |
| Boundaries | The caller has concerns relating to professional boundaries. |
| Dissatisfaction with care | The caller reports that a patient or family member has expressed dissatisfaction with care. |
| Ending a doctor patient relationship | The caller is inquiring about ending the doctor patient relationship after either (1) a recent encounter has threatened mutual trust or (2) because the physician is retiring or reducing the size of their practice. |
| Opioids | The caller has questions about the use of opioids in their practice during the COVID-19 pandemic. |
| Patient dishonesty | The caller has concerns because one of their patients has been dishonest (e.g. patient intentionally misled physician about recent international travel or contact with a COVID-positive individual). |
| Patient refusing IPAC measures | The caller is concerned about a patient who has refused IPAC (e.g. masks, handwashing, answering questions, temperature checks). |
| Requests for physician note | The caller is asking about a pandemic-related note for patients, including documentation for schools, daycare, work, workplace accommodation requests, etc. |
| Visitor restrictions | The caller’s concern relates to visitor restrictions due to the pandemic (e.g., hospital or long-term care). |
| **Theme 4. Physician obligation and rights:** Family physicians seeking guidance related to their obligations and rights in relation to the pandemic. | |
| **Code** | **Definition** |
| Colleague refusing IPAC measures | A colleague has refused to practice what the caller feels are appropriate infection prevention and control measures |
| Duty of care | The caller has some concern over their obligation to care for a patient due to practice changes in the pandemic (e.g. "Am I required to provide care for this person or in these circumstances?") |
| Duty to report | The caller has concerns about a patient's or another professional's behaviour and is considering their duty to report to an authority (e.g. public health). |
| Exams and certifications | The caller has questions about exams and certifications that have been impacted by the pandemic. |
| Non-resident patients | The caller has questions about providing care for patients who are not Canadian residents. |
| Physician's personal health risk | The caller has a health condition that puts them or an immediate family members at increased risk for COVID, which is central to their concern. |
| Physician privacy | The caller has a concern related to their own or another physician’s privacy, e.g., COVID test results / symptoms. |
| Physician stress/burnout | The caller states that they are under a high level of stress or feeling burnt out due to the pandemic |
| Physician travel | The caller plans to travel out of province or country, or has recently done so or is being asked to do so, including concerns about testing and isolation requirements |
| Scope of practice | The caller has concern regarding changes in their scope of practice due to COVID |
| Work-life balance | The caller expresses concern related to their ability to keep up with their work due to family responsibilities. |
| **Theme 5. Public health matters:** Family physicians seeking guidance related to public health. | |
| **Code** | **Definition** |
| Advocacy requests | The caller is asking what the CMPA is doing to advocate for physicians during the pandemic. |
| Confusion and inconsistency / interpretation of guidelines | The caller has questions about how to interpret or adhere to public health guidelines for themselves or on behalf of a patient. |
| COVID exposure or transmission | The caller has concerns regarding exposure and transmission of COVID-19 in their clinic or healthcare facility |
| Public health role | The caller has concerns around a matter related to their public health role (e.g. public health advisor, administrator, member on hospital’s pandemic committees) |
| Public statements and advice to non-patients | The caller has concerns about statements they have made to the public related to COVID-19. |
| Reopening clinics for in-person care | The caller has questions or concerns related to re-opening offices to provide in-person, elective patient care or procedures |
| Self-isolation | Concerns around self-isolation, either on the part of the member calling or a patient or colleague |
| **Theme 6. Virtual care:** Family physicians seeking guidance related to telemedicine and remote care. | |
| **Code** | **Definition** |
| Cross-border care | The caller has concerns related to providing health care across international borders during the pandemic. |
| Information management | The caller has questions related to managing patients' confidential information due to practice changes during the pandemic. |
| Inter-provincial care | The caller has concerns related to inter-provincial care during the pandemic. |
| Telemedicine | The caller has questions about the use of telemedicine or remote care. |
| Telemedicine – Standard of care | The caller has a concern about insuring an appropriate standard of care via telemedicine. |
